# Molecular classification of human papillomavirus-positive cervical cancers based on immune signature enrichment

**DOI:** 10.3389/fpubh.2022.979933

**Published:** 2022-09-20

**Authors:** Guanghui Song, Jiangti Luo, Shaohan Zou, Fang Lou, Tianfang Zhang, Xiaojun Zhu, Jianhua Yang, Xiaosheng Wang

**Affiliations:** ^1^Department of Gynecology and Obstetrics, Sir Run Run Shaw Hospital, Medical School of Zhejiang University, Hangzhou, China; ^2^Biomedical Informatics Research Lab, School of Basic Medicine and Clinical Pharmacy, China Pharmaceutical University, Nanjing, China; ^3^Cancer Genomics Research Center, School of Basic Medicine and Clinical Pharmacy, China Pharmaceutical University, Nanjing, China; ^4^Big Data Research Institute, China Pharmaceutical University, Nanjing, China; ^5^Department of Medical Oncology, Sir Run Run Shaw Hospital, Medical School of Zhejiang University, Hangzhou, China; ^6^Department of Rehabilitation Medicine, The First Affiliated Hospital, Zhejiang University School of Medicine, Hangzhou, China; ^7^Department of Gynecology and Obstetrics, Women's Hospital, School of Medicine, Zhejiang University, Hangzhou, China

**Keywords:** human papillomavirus-positive cervical cancer, immunological classification, machine learning, multi-omics analysis, immunotherapy

## Abstract

**Background:**

Human papillomavirus-positive (HPV+) cervical cancers are highly heterogeneous in clinical and molecular characteristics. Thus, an investigation into their heterogeneous immunological profiles is meaningful in providing both biological and clinical insights into this disease.

**Methods:**

Based on the enrichment of 29 immune signatures, we discovered immune subtypes of HPV+ cervical cancers by hierarchical clustering. To explore whether this subtyping method is reproducible, we analyzed three bulk and one single cell transcriptomic datasets. We also compared clinical and molecular characteristics between the immune subtypes.

**Results:**

Clustering analysis identified two immune subtypes of HPV+ cervical cancers: Immunity-H and Immunity-L, consistent in the four datasets. In comparisons with Immunity-L, Immunity-H displayed stronger immunity, more stromal contents, lower tumor purity, proliferation potential, intratumor heterogeneity and stemness, higher tumor mutation burden, more neoantigens, lower levels of copy number alterations, lower DNA repair activity, as well as better overall survival prognosis. Certain genes, such as *MUC17, PCLO*, and *GOLGB1*, showed significantly higher mutation rates in Immunity-L than in Immunity-H. 16 proteins were significantly upregulated in Immunity-H vs. Immunity-L, including Caspase-7, PREX1, Lck, C-Raf, PI3K-p85, Syk, 14-3-3_epsilon, STAT5-α, GATA3, Src_pY416, NDRG1_pT346, Notch1, PDK1_pS241, Bim, NF-kB-p65_pS536, and p53. Pathway analysis identified numerous immune-related pathways more highly enriched in Immunity-H vs. Immunity-L, including cytokine-cytokine receptor interaction, natural killer cell-mediated cytotoxicity, antigen processing and presentation, T/B cell receptor signaling, chemokine signaling, supporting the stronger antitumor immunity in Immunity-H vs. Immunity-L.

**Conclusion:**

HPV+ cervical cancers are divided into two subgroups based on their immune signatures' enrichment. Both subgroups have markedly different tumor immunity, progression phenotypes, genomic features, and clinical outcomes. Our data offer novel perception in the tumor biology as well as clinical implications for HPV+ cervical cancer.

## Introduction

The human papillomavirus (HPV) infection is a leading etiology for cervical cancer, the most common gynecological malignancy (1). Among 15 high-risk HPV strains, HPV16 and HPV18 infection are associated with 70% of HPV-related cervical cancers (2). Squamous cell carcinomas and adenocarcinomas constitute 90 and 10% of cervical cancers, respectively. Furthermore, cervical squamous cell carcinomas are predominantly HPV-positive (HPV+), compared to 25% of cervical adenocarcinomas being HPV-negative (HPV-) (3). Prior investigations have demonstrated the high heterogeneity of cervical cancer (4). For example, The Cancer Genome Atlas (TCGA) network classified cervical cancers into four subtypes, namely HPV clade A9, A7, HPV-negative, and other, based on the HPV status (4). TCGA also identified three subtypes of cervical cancers: hormone, epithelial-mesenchymal transition (EMT), and PI3K-AKT, based on protein expression profiles (4). By an integrative analysis of multi-omics profiles, including mRNA and microRNA expression, copy number alterations (CNAs), and DNA methylation, TCGA uncovered three subtypes of cervical cancer: keratin-high squamous, keratin-low squamous, and adenocarcinoma-rich (4). Li et al. (5) identified six cervical cancer subtypes by consensus clustering, of which two immune clusters-associated gene signatures were valuable prognostic factors.

Surgery, radiotherapy, and chemotherapy are the major therapeutic approaches for cervical cancer to date. However, these approaches often provide only limited efficiency for late stage of cervical cancers (6). Immunotherapies, particularly immune checkpoint inhibitors (ICIs), have recently demonstrated successes in treating various malignancies, including melanoma, lung cancer, bladder cancer, head and neck cancer, kidney cancer, liver cancer, breast cancer, cervical cancer, prostate cancer, and the cancers with high tumor mutation loads or mismatch repair deficiency (dMMR). Studies have shown that ICIs may result in the remission of virus infection-associated malignancies, including HPV infection-associated cervical (7) and head and neck (8) cancers. Nevertheless, the response rates to ICIs are relatively low, with currently only around 20% of cancer patients responding to ICIs (9). Thus, identifying the determinants of immunotherapy responses may aid in improving the cancer immunotherapeutic efficiency. Accumulating evidence has demonstrated that, certain molecular features, e.g., PD-L1 expression (10), high tumor mutation burden (TMB) (11), and dMMR (12), are associated with better immunotherapeutic responsiveness. In addition, the “hot” tumors with strong immune infiltration likely respond better to immunotherapy than the “cold” tumors with weak immune infiltration (13). Thus, differentiating “hot” tumors from “cold” tumors may facilitate the optimal selection of cancer patients for immunotherapy. With the recent emergence of massive multi-omics data in cancer research, many algorithms have been developed to uncover “hot” and “cold” tumor subtypes, such as unsupervised machine learning algorithms (14–16).

In the present study, we identified immune-related subtypes of HPV+ cervical cancers based on the enrichment of 29 immune signatures by unsupervised machine learning. This analysis identified two subtypes of HPV+ cervical cancers, which exhibited high and low enrichment of immune signatures, respectively, reproducibly in four different cohorts. Furthermore, we comprehensively compared clinical and molecular characteristics between both subtypes of HPV+ cervical cancers. This study could offer new understanding of the tumor biology as well as clinical implications for the management of HPV+ cervical cancer.

## Methods

### Datasets

We downloaded multi-omics data for cervical squamous cell carcinoma and endocervical adenocarcinoma (CESC) in TCGA, termed TCGA-CESC (4), from the genomic data commons (GDC) data portal (https://portal.gdc.cancer.gov/). These multi-omics data included mRNA gene expression profiles (normalized by RSEM), somatic mutations (“maf” file), somatic copy number alterations (SCNAs) (“SNP6” files), protein expression profiles (Reverse Phase Protein Array (RPPA), normalized), and clinical data. We obtained a CESC transcriptomic dataset (SGCX) from a prior publication (17), and a CESC transcriptomic dataset (GSE29570) (18) from the NCBI gene expression omnibus (GEO) (https://www.ncbi.nlm.nih.gov/geo/). We also downloaded a single-cell transcriptomic dataset (GSE171894) for CESC from the NCBI GEO. A description of these datasets is shown in Supplementary Table S1.

### Single-sample gene set enrichment analysis

We employed the single-sample gene set enrichment analysis (ssGSEA) (19) to assess the enrichment levels of immune signatures, biological processes, and pathways in tumors. The ssGSEA evaluates the enrichment score of a gene set in a sample based on the expression profiles of their marker or pathway genes (19). The marker or pathway genes of immune signatures, pathways, and biological processes we analyzed are listed in Supplementary Table S2. The “GSVA” R package (19) was utilized to perform the ssGSEA.

### Clustering analysis

We identified immune-related subtypes of HPV+ cervical cancers by hierarchical clustering based on the enrichment scores of 29 immune signatures. These immune signatures included antigen presenting cell (APC) co-inhibition, APC co-stimulation, B cells, cytokine and cytokine receptor (CCR), CD8+ T cells, immune checkpoint, cytolytic activity, dendritic cells (DCs), activated DCs (aDCs), immature DCs (iDCs), plasmacytoid dendritic cells (pDCs), human leukocyte antigen (HLA), inflammation-promoting, macrophages, mast cells, major histocompatibility complex (MHC) class I, neutrophils, natural killer (NK) cells, parainflammation, T cell co-inhibition, T cell co-stimulation, T helper cells, T follicular helper (Tfh), T helper 1 (Th1) cells, T helper 2 (Th2) cells, tumor infiltrating lymphocytes (TILs), regulatory T (Treg) cells, Type I interferon (IFN) response, and Type II IFN response. Before clustering, we performed normalization of the ssGSEA scores by Z-score and transformed them into distance matrices by the R function “dist” with the parameter: method = “euclidean.” The hierarchical clustering was implemented with the function “hclust” in the R package “Stats” with the parameters: method = “ward.D2” and members = NULL.

### Class prediction

We employed the Random Forest (RF) algorithm to predict the immune-related subtypes of HPV+ cervical cancers based on the ssGSEA scores of immune signatures. The number of trees was 500 and the predictors were the 29 immune signatures in the RF. The accuracy and weighted F-score were reported as the prediction performance. The RF algorithm was implemented by using the “randomForest” R package (20).

### Survival analysis

We utilized the Kaplan-Meier (K-M) model (21) to compare overall survival (OS) and disease-free survival (DFS) time between the immune subtypes of HPV+ cervical cancers. K-M curves were plotted to display the survival time differences, and the log-rank test was utilized to assess whether the survival time differences were significant with a threshold of *P* < 0.05.

### Calculation of TMB, SCNA, intratumor heterogeneity, immune scores, stromal content, and tumor purity in tumors

We determined a tumor's TMB as the total number of its somatic mutations. With the input of “SNP6” files, we employed GISTIC2 (22) to calculate G-scores in tumors. The G-score represents the amplitude of the SCNA and the frequency of its occurrence across a class of samples (22). We utilized the DITHER algorithm (23) to score ITH, which measures ITH based on both somatic mutation and SCNA profiles. The ESTIMATE algorithm (24) was used to calculate immune scores, stromal scores, and tumor purity for bulk tumors, which represent immune infiltration levels, stromal contents, and proportions of tumor cells in bulk tumors.

### Pathway analysis

To identify pathways more enriched in one subgroup relative to another subgroup, we first identified upregulated genes in the subgroup vs. another subgroup by Student's *t*-test with a threshold of false discovery rate (FDR) < 0.05 and fold change (FC) > 1.5. By inputting the upregulated genes into the GSEA web tool (25), we obtained the KEGG (26) pathways more enriched in the subgroup with a threshold of FDR < 0.05.

### Single-cell RNA sequencing data analysis

We analyzed a scRNA-seq transcriptomic dataset (GSE171894) for HPV+ cervical cancers. The gene expression values in single cells were normalized by transcripts per million (TPM). We performed hierarchical clustering of HPV+ cervical cancer single cells based on immune signatures' scores to identify their subtypes. In addition, the single-cell consensus clustering (SC3) method (27) were utilized to cluster single cells in each immune subtype, respectively.

### Statistical analysis

We used the two-tailed Student's *t*-test to compare two classes of normally-distributed data, including gene expression values, protein expression values, and the enrichment ratios of two immune signatures. The ratios were the log2-transformed values of the average expression levels of all marker genes in an immune signature over those of all marker genes in another immune signature. When comparing two classes of non-normally distributed data, including ssGSEA scores, TMB, neoantigens, ITH, immune scores, stromal scores, and tumor purity, we used the one-tailed Mann–Whitney *U*-test. We used the Spearman method to assess the correlation between pathway enrichment scores (ssGSEA scores) and immune scores. The Fisher's exact test was utilized to analyze contingency tables. To adjust for *P*-values in multiple tests, we calculated FDR by using the Benjamini and Hochberg method (28). We performed all statistical analyses and visualizations with the R programming (version 3.6.1).

## Results

### Unsupervised clustering identifies two immune subtypes of HPV+ cervical cancer

**Figure 1:**
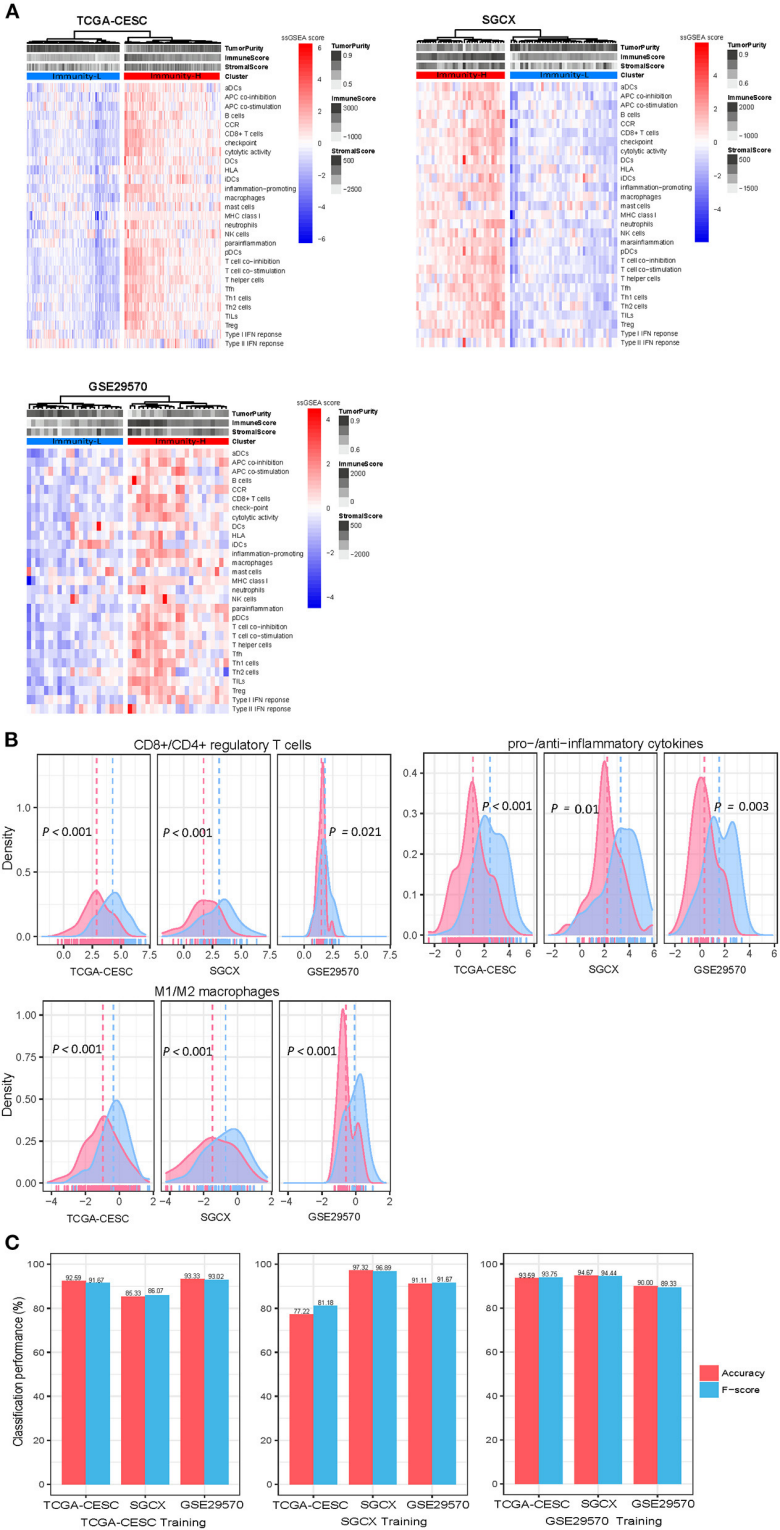
Clustering analysis identifies two immune subtypes of HPV+ cervical cancer. **(A)** Based on the enrichment levels of 29 immune signatures, hierarchical clustering identifies two immune subtypes: Immunity-H and Immunity-L, which have high and low immunity, respectively, consistently in three datasets. **(B)** Immunity-H has significantly higher ratios of immunostimulatory to immunosuppressive signatures than Immunity-L. The two-tailed Student's *t*-test *P*-values are shown. **(C)** Prediction of the immune subtypes of HPV+ cervical cancer by Random Forest based on the enrichment scores of 29 immune signatures. The 10-fold cross-validation results in the training set and prediction results in the other test sets are shown.

Based on the enrichment scores of 29 immune signatures, we identified two immune subtypes of HPV+ cervical cancer, consistently in three datasets (TCGA-CESC, GSE29570, and SGCX) (Figure 1A). Both subtypes had high and low enrichment of these immune signatures, termed Immunity-H and Immunity-L, respectively. Of note, both immunostimulatory signatures (such as CD8+ T cells, T cell co-stimulation, APC co-stimulation, cytolytic activity, and NK cells) and immunosuppressive signatures (such as CD4+ regulatory T cells, APC co-inhibition, T cell co-inhibition, and immune checkpoint molecules) showed significantly higher enrichment levels in Immunity-H than in Immunity-L (Figure 1A). Nevertheless, the ratios of immunostimulatory to immunosuppressive signatures (CD8+/CD4+ regulatory T cells, pro-/anti-inflammatory cytokines, and M1/M2 macrophages) were significantly higher in Immunity-H than in Immunity-L (two-tailed Student's *t*-test, *P* < 0.05) (Figure 1B). Collectively, these results supported that Immunity-H had more active antitumor immune responses than Immunity-L.

To explore whether this subtyping method is predictable, we used one of the three datasets as the training set and the other two datasets as test sets in turn to predict both subtypes with RF. The 10-fold CV accuracies and weighted F-scores in the training sets were >77%. The prediction accuracies and weighted F-scores in test sets were all above 80% (Figure 1C). These results support that the subtyping is predictable and robust.

### The immune subtypes of HPV+ cervical cancer have significantly different clinical and molecular characteristics

**Figure 2:**
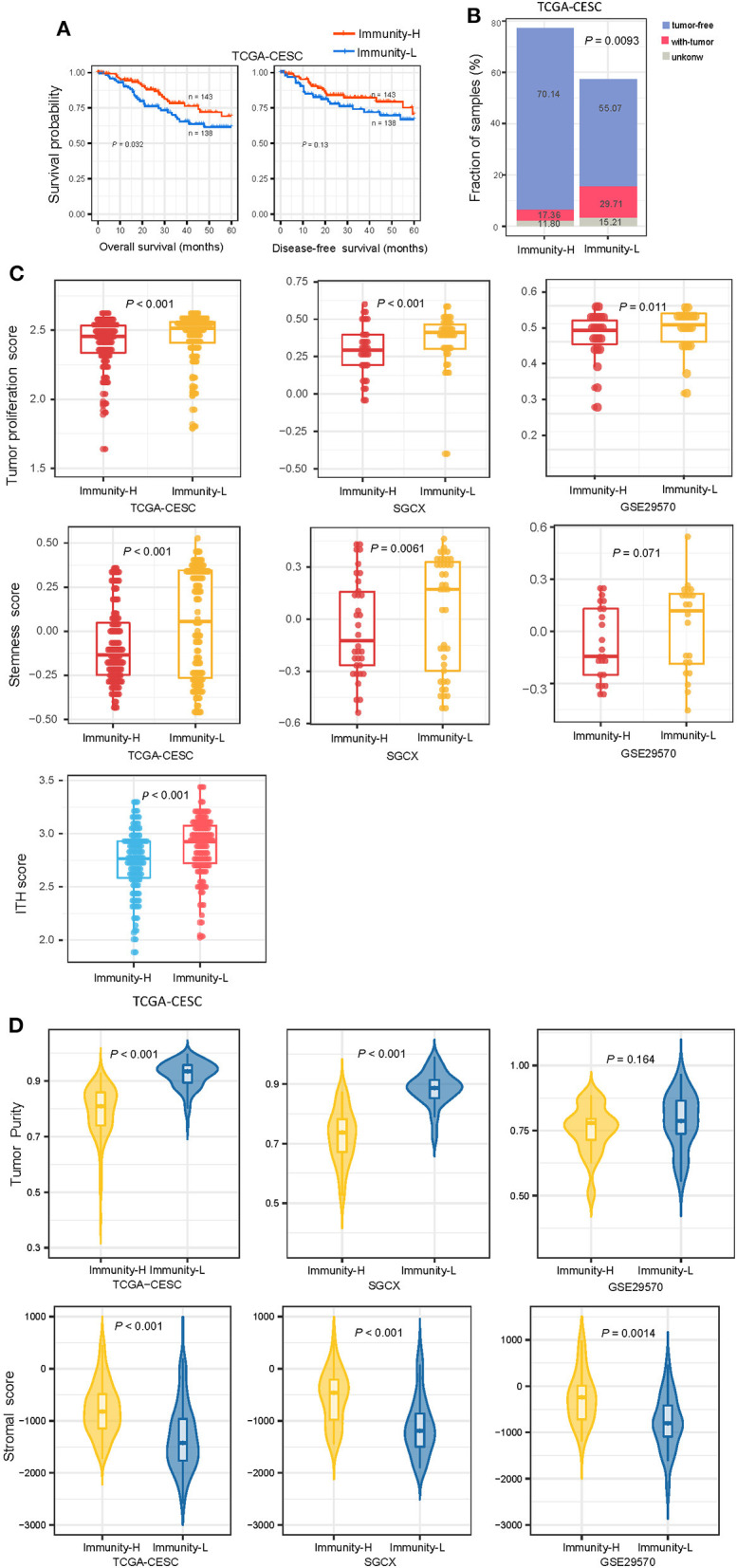
Comparisons of clinical and molecular features between the immune subtypes of HPV+ cervical cancer. **(A)** Kaplan–Meier curves showing 5-year overall survival and disease-free survival time differences between the immune subtypes. The log-rank test *P*-values are shown. **(B)** The proportion of tumor-free patients is significantly higher in Immunity-H than in Immunity-L The Fisher's exact-test *P*-value is shown. **(C)** Immunity-H displays significantly lower enrichment scores of tumor proliferation, stemness, and ITH than in Immunity-L. **(D)** Comparisons of tumor purity and stromal content between the immune subtypes. The one-tailed Mann–Whitney *U*-test *P*-values are shown in **(C,D)**. ITH, intratumor heterogeneity.

We compared 5-year OS and DFS prognosis between both subtypes in TCGA-CESC, which had related data available. Kaplan-Meier curves showed that Immunity-H had significantly higher OS rates than Immunity-L (log-rank test, *P* < 0.05) (Figure 2A). We found that the proportion of tumor-free patients was significantly higher in Immunity-H than in Immunity-L (Fisher's exact test, *P* = 0.009; odds ratio (OR) = 0.46) (Figure 2B). It confirmed that Immunity-H had a better prognosis than Immunity-L. We further compared several phenotypic or molecular features associated with tumor progression, including tumor proliferation, stemness, and ITH. Of note, these features were more enriched in Immunity-L than in Immunity-H (*P* < 0.05) (Figure 2C). Furthermore, tumor purity was likely higher in Immunity-L than in Immunity-H, while stromal content was more enriched in Immunity-H than in Immunity-L (Figure 2D).

**Figure 3:**
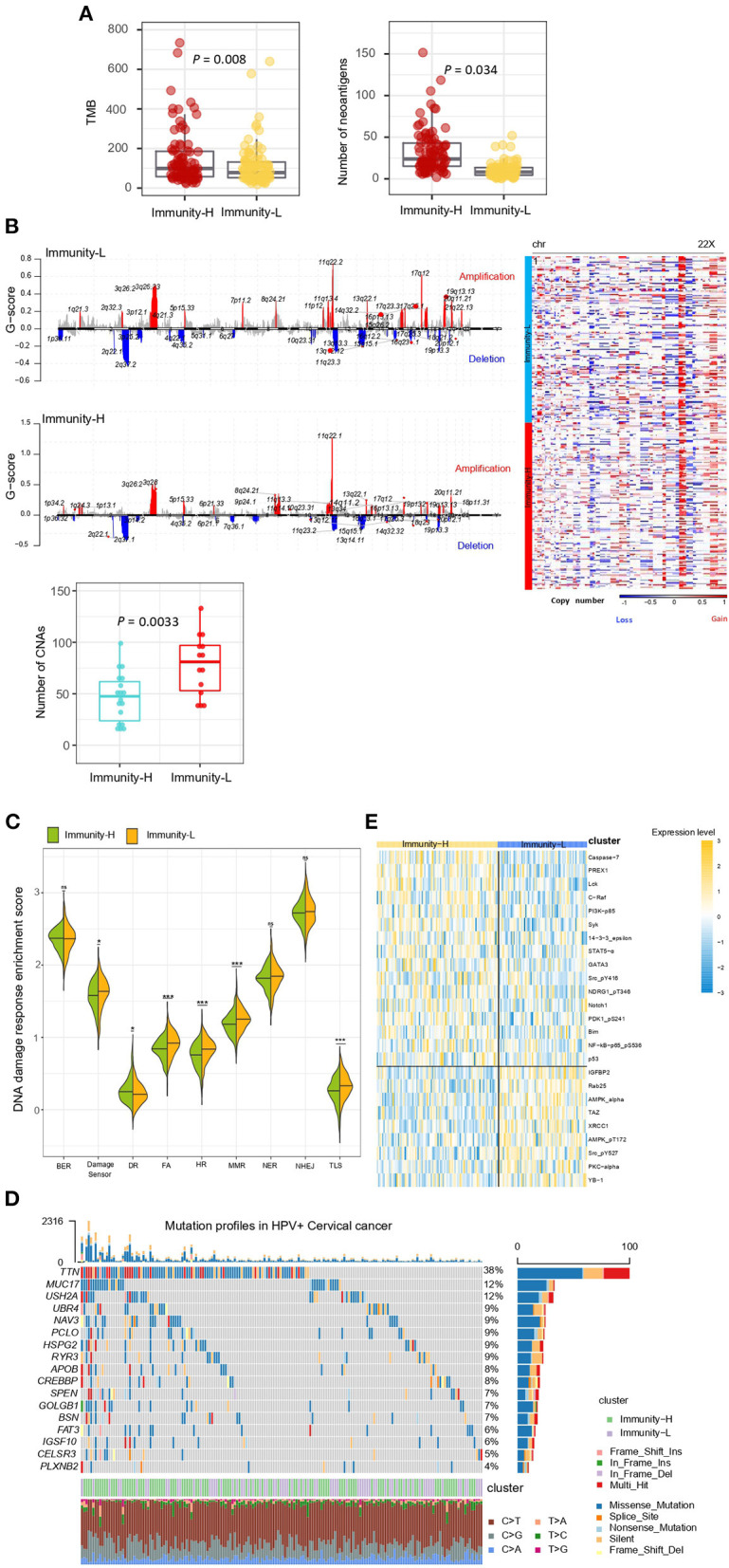
Comparisons of somatic mutation, copy number alteration, and protein expression profiles between the immune subtypes of HPV+ cervical cancer**. (A)** Immunity-H has significantly higher tumor mutation burden (TMB) and more predicted neoantigens than Immunity-L. **(B)** Immunity-H has significantly lower extent of copy number alterations than Immunity-L. **(C)** Six of the nine major DNA damage response (DDR) pathways shows significantly higher enrichment scores in Immunity-L than in Immunity-H. BER, base excision repair. DR, direct damage reversal/repair. NER, nucleotide excision repair. MMR, mismatch repair. FA, Fanconi Anemia HR, homologous recomination. NHEJ, non-homologous end joining. TLS, translesion synthesis. **(D)** The mutation profiles of the genes in HPV+ Cervical cancer, which have significantly higher mutation rates in Immunity-L than in Immunity-H. **(E)** Heatmap shows 25 proteins differentially expressed between Immunity-L and Immunity-H (two-tailed Student's *t*-test, FDR < 0.05). The one-tailed Mann–Whitney *U*-test *P*-values are shown in **(A–C)**. *, *P* < 0.05; **, *P* < 0.01; ***, *P* < 0.001; ^ns^, *P* ≥ 0.05.

Immunity-H had significantly higher TMB (*P* = 0.008) and thus more predicted neoantigens (29) (*P* = 0.034) than Immunity-L (Figure 3A). In contrast, Immunity-H had significantly lower levels of tumor aneuploidy, also known as CNA, than Immunity-L, as evidenced by markedly lower CNA scores (30) and G-scores of copy number amplifications and deletions (Figure 3B). These results conform to previous findings that TMB and tumor aneuploidy correlate positively and negatively with antitumor immune responses, respectively (31). Furthermore, we compared the enrichment of nine major DNA damage response (DDR) pathways between both subtypes. These pathways included base excision repair, mismatch repair, nucleotide excision repair, the Fanconi anemia (FA) pathway, homology-dependent recombination, non-homologous DNA end joining, direct damage reversal/repair, translesion DNA synthesis, and damage sensor (30). Of note, six of the nine pathways displayed significantly higher enrichment in Immunity-L than in Immunity-H (*P* < 0.05) (Figure 3C). It indicates that Immunity-L has a stronger DDR than Immunity-H. It is justified since Immunity-L displays higher genomic instability than Immunity-H.

We found 17 genes showing significantly higher mutation rates in Immunity-L than in Immunity-H (Fisher's exact test, *P* < 0.05; OR > 2), but no gene having significantly higher mutation rates in Immunity-H versus Immunity-L (Figure 3D). These genes included *PCLO, TTN, IGSF10, MUC17, FAT3, APOB, MED1, ODZ2, UBR4, CREBBP, GOLGB1, USH2A, ABCA12, HSPG2, SPEN, CELSR3, MDC1, PLXNB2, MLL3, NAV3, BSN*, and *RYR3*. *CREBBP* (CREB binding protein) encodes a transcription factor that is involved in the transcriptional coactivation of many transcription factors. This gene had a mutation rate of 10% in Immunity-L vs. 2.8% in Immunity-H (*P* = 0.01). Its mutations have been associated with antitumor immunosuppression (32), consistent with our result. *MUC17* (mucin 17, cell surface associated) encodes a member of membrane-bound mucins, which are involved in tumor immunomodulation (33). This gene had a mutation rate of 11.6% in Immunity-L vs. 2.1% in Immunity-H (*P* = 0.002). *PCLO* (piccolo presynaptic cytomatrix protein) encodes a protein which is part of the presynaptic cytoskeletal matrix. This gene's mutations have been associated with alterations of tumor immunity (34). *GOLGB1* (golgin B1) encodes a protein located in Golgi apparatus and endoplasmic reticulum-Golgi intermediate compartment. Previous studies have shown that *GOLGB1* mutations were more prevalent in “cold” tumors (35), consistent with our analysis revealing its higher mutation rate in Immunity-L vs. Immunity-H (10.1 vs. 2.8%; *P* = 0.014).

We compared the expression levels of 226 proteins between both subtypes and found 25 differentially expressed proteins (DEPs) (two-tailed Student's *t*-test, *P* < 0.05) (Figure 3E). Among the DEPs, 16 were upregulated in Immunity-H, including Caspase-7, PREX1, Lck, C-Raf, PI3K-p85, Syk, 14-3-3_epsilon, STAT5-α, GATA3, Src_pY416, NDRG1_pT346, Notch1, PDK1_pS241, Bim, NF-kB-p65_pS536, and p53. Of note, many of these proteins play a role in the positive regulation of antitumor immune responses. For example, Caspase-7 is positively associated with antitumor immune responses by the regulation of apoptosis (36). PREX1 acts as a guanine nucleotide exchange factor for the RHO family of small GTP-binding proteins (RACs) to promote antitumor immune responses (37). Lck incites antitumor immune responses by regulating T cell development (38). Syk as a tumor suppressor has a role in driving antitumor immune responses (39). STAT5 has also been shown to promote antitumor immunity (40). Among the DEPs, nine were downregulated in Immunity-H, including IGFBP2, Rab25, AMPK-α, TAZ, XRCC1, AMPK_pT172, Src_pY527, PKC-alpha, and YB-1. It suggests that these proteins have a negative association with antitumor responses. Previous studies have shown that IGFBP2 upregulation can drive the growth of tumors and promote antitumor immunosuppression (41), supporting our result. TAZ is a component of the Hippo signaling pathway, which may promote tumor immune evasion (15, 42). It is in agreement with our finding that this protein is downregulated in Immunity-H. YB-1 is a cold shock domain protein implicated in numerous cellular processes, whose upregulation drives cancer proliferation and immune evasion (43). Again, it is consistent with our finding. XRCC1 is involved in DNA repair, whose upregulation indicates a stronger DNA repair capacity. It is in line with the previous finding that the DDR pathways are more enriched in Immunity-L than in Immunity-H. A previous study revealed that AMP-activated protein kinase (AMPK) plays a pivotal role in regulating the response to immune-checkpoint blockade, indicating that AMPK agonists may promote the efficacy of immunotherapy (44). This previous finding supports our result that AMPK is upregulated in “cold” tumors.

### Identification of pathways enriched in the immune subtypes of HPV+ cervical cancer

**Figure 4:**
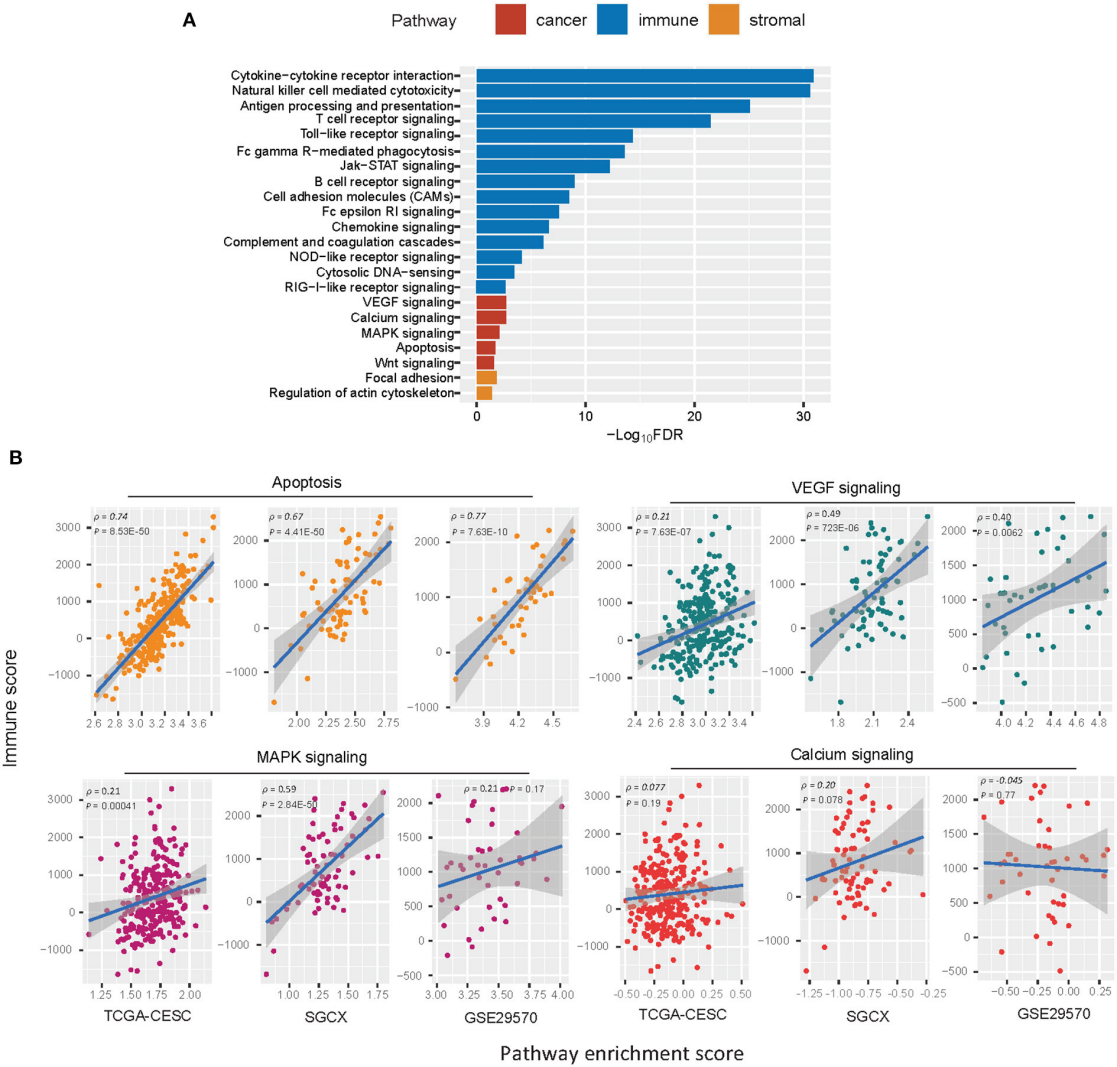
KEGG pathways more enriched in Immunity-H vs. Immunity-L. **(A)** The cancer, immune, and stroma-associated pathways showing significantly higher enrichment in Immunity-H (FDR <0.05). FDR, false discovery rate. **(B)** Spearman correlations between the enrichment scores of the cancer-associated pathways upregulated in Immunity-H and immune scores in HPV+ cervical cancer.

GSEA (25) identified numerous KEGG pathways more enriched in Immunity-H vs. Immunity-L. As expected, many immune-relevant pathways were included in the list, including cytokine-cytokine receptor interaction, natural killer cell-mediated cytotoxicity, antigen processing and presentation, T cell receptor signaling, Toll-like receptor signaling, Fc gamma R-mediated phagocytosis, Jak-STAT signaling pathway, B cell receptor signaling, Fc epsilon RI signaling, NOD-like receptor signaling, cytosolic DNA-sensing, cell adhesion molecules (CAMs), chemokine signaling, RIG-I-like receptor signaling, and complement and coagulation cascades (Figure 4A). Besides, some stromal pathways were also included in the list, such as regulation of actin cytoskeleton and focal adhesion. It is consistent with the previous finding that Immunity-H has higher stromal content than Immunity-L. In addition, several cancer-associated pathways were more enriched in Immunity-H vs. Immunity-L, including apoptosis, VEGF signaling, calcium signaling, MAPK signaling, and Wnt signaling pathways. It indicates a positive association between the enrichment of these pathways and antitumor immune responses. Indeed, we found that the enrichment scores of most these pathways were positively correlated with immune scores in HPV+ cervical cancer (Spearman correlation, *P* < 0.05) (Figure 4B). These results conform to previous findings of the significant positive association between these pathways' enrichment and antitumor immunity (45). We also identified certain pathways more enriched in Immunity-L vs. Immunity-H, most of which were metabolism relevant, such as maturity onset diabetes of the young, metabolism of xenobiotics by cytochrome P450, drug metabolism - cytochrome P450, retinol metabolism, O-Glycan biosynthesis, starch and sucrose metabolism, and glycosphingolipid biosynthesis - lacto and neolacto series.

### Unsupervised clustering identifies two immune subtypes of HPV+ cervical cancer single cells

**Figure 5:**
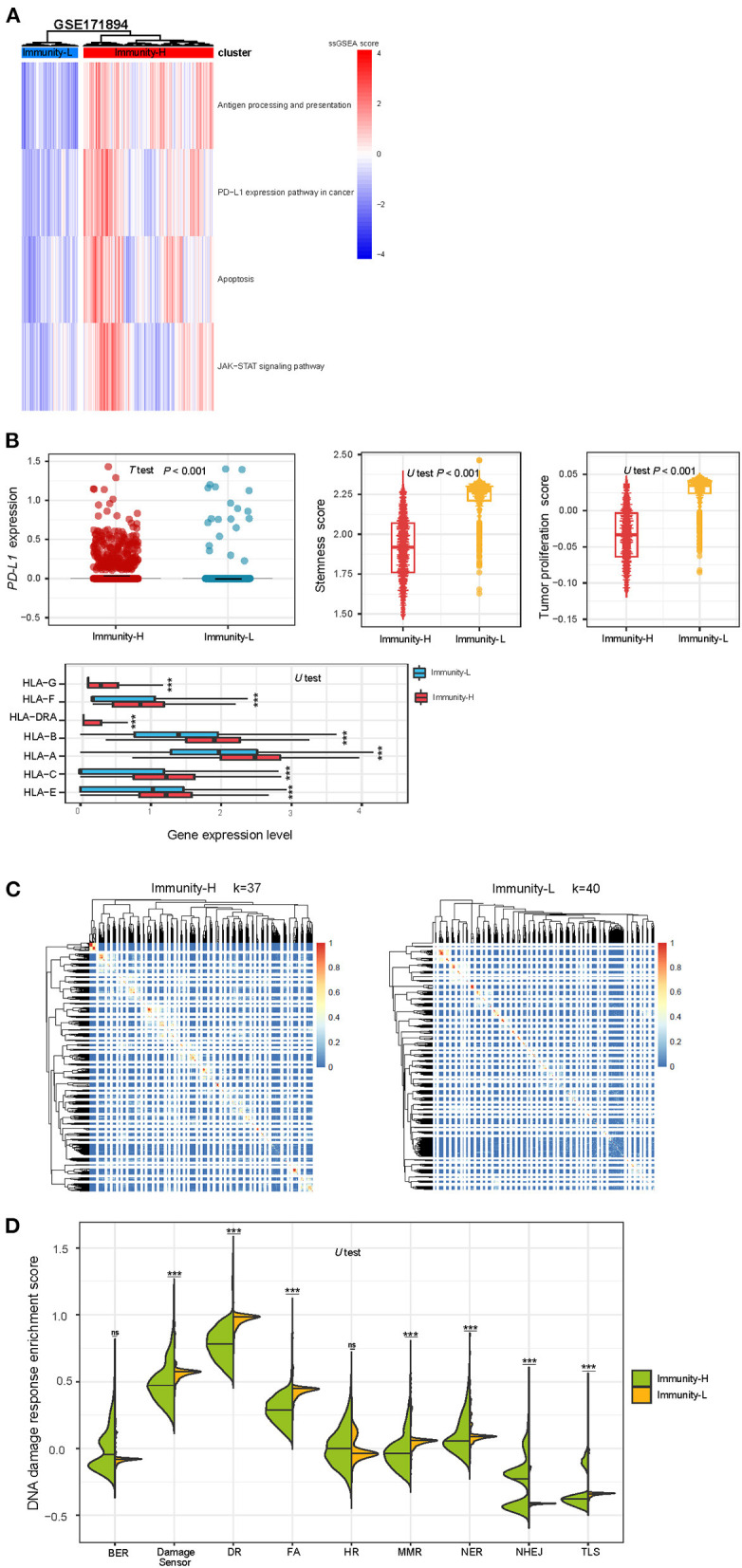
Validation of the immune-specific subtyping method in HPV+ cervical cancer single cells. **(A)** Hierarchical clustering of 3,356 cancer cells from 2 HPV+ cervical cancer patients based on the enrichment scores of four immune-related pathways. **(B)** The expression levels of *PD-L1* and many HLA genes are significantly higher in Immunity-H than in Immunity-L cancer single cells, while tumor stemness and proliferation scores are significantly lower in Immunity-H cancer single cells. **(C)** Consensus clustering of single cells in Immunity-H and Immunity-L by SC3 (27) identifying 37 and 40 cell clusters, respectively. **(D)** Seven of the nine major DNA damage response pathways shows significantly higher enrichment scores in Immunity-L than in Immunity-H. BER, base excision repair. DR, direct damage reversal/repair. NER, nucleotide excision repair. MMR, mismatch repair. FA, Fanconi Anemia. HR, homologous recomination. NHEJ, non-homologous end joining. TLS, translesion synthesis.

Likewise, we performed a hierarchical clustering of HPV+ cervical cancer single cells in a scRNA-seq dataset (GSE171894), which was a gene expression profiling in 3,356 cancer cells from 2 HPV+ cervical cancer patients. Because this clustering analysis was performed in cancer single cells, we used four immune-related pathways which are expressed in cancer cells themselves. These pathways included antigen processing and presentation, JAK-STAT signaling, apoptosis, and PD-L1 expression pathway in cancer. Similarly, we identified two clusters of these cancer single cells, also termed Immunity-H and Immunity-L, respectively. Immunity-H and Immunity-L displayed high and low enrichment scores of these pathways (Figure 5A). As expected, Immunity-H had significantly higher *PD-L1* expression levels than Immunity-L (*P* < 0.001); Immunity-H also showed significantly higher expression levels of many HLA genes than Immunity-L (*P* < 0.001); Immunity-L displayed significantly stronger proliferation and stemness signatures than Immunity-H (*P* < 0.001) (Figure 5B). We further performed the consensus clustering of Immunity-H and Immunity-L single cells by SC3 (27), respectively. The clustering identified 37 and 40 cell clusters in Immunity-H and Immunity-L single cells, respectively (Figure 5C). It suggests a higher heterogeneity of cancer cells in Immunity-L compared to Immunity-H, consistent with the result from bulk tumors. Immunity-L had significantly higher enrichment of the DDR pathways than Immunity-H (Figure 5D). These results are consistent with those in bulk tumors.

## Discussion

**Figure 6:**
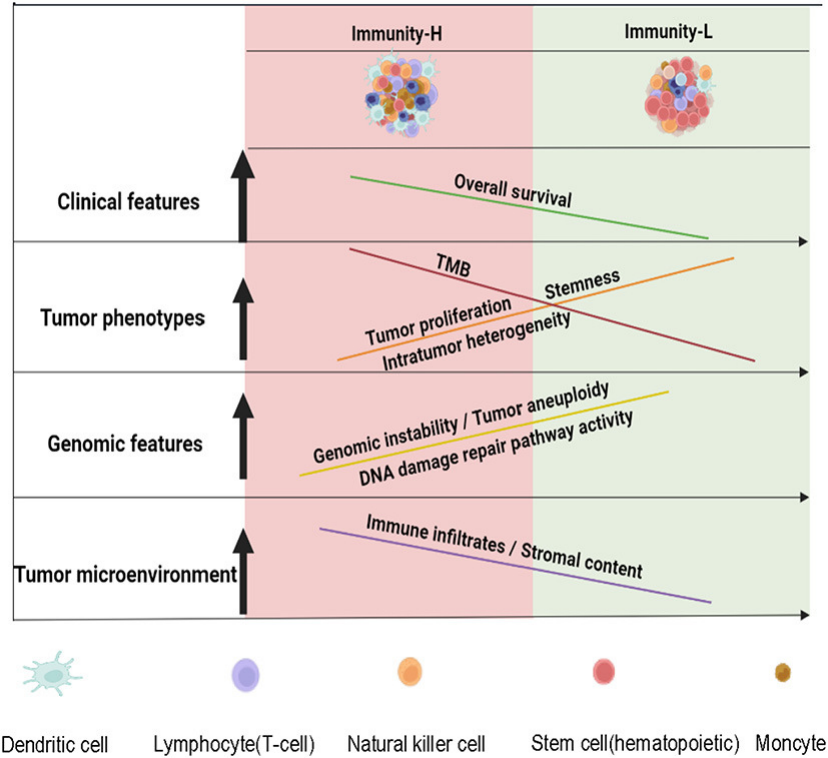
A schematic illustration to summarize the molecular and clinical features of both immune subtypes of HPV+ cervical cancer. HPV+ cervical cancer can be classified into two subtypes with high and low immunity, respectively. The low immunity is associated with tumor progressive phenotypes, unfavorable prognosis, and genomic instability in HPV+ cervical cancer. The figure was created with BioRender.com.

The tumor immune microenvironment (TIME) plays crucial roles in determining cancer prognosis and therapy responses (46). Meanwhile, the TIME heterogeneity is prevalent across tumors (47). Hence, a classification of HPV+ cervical cancer based on the TIME would have potential clincal values in risk stratification and effective treatment of this disease. To this end, we identified two immune subtypes of HPV+ cervical cancer based on the enrichment of 29 immune signatures by unsupervised clustering. We demonstrated the reproducibility and predictability of this subtyping method by analyzing four different datasets, including three bulk tumor datasets and one single cell dataset. Compared to Immunity-L, Immunity-H showed higher immunity, more stromal contents, lower tumor purity, lower tumor proliferation potential, stemness, and ITH, higher TMB, more neoantigens, lower levels of CNAs, lower DDR activity, as well as better overall survival prognosis (Figure 6). Our analysis confirmed that “hot” tumors have a stronger antitumor immunity and thus more favorable prognosis vs. “cold” tumors (15, 45, 48). Previous studies have shown that TMB and CNAs correlate positively and negatively with antitumor immune response, respectively (31). This is consistent with our results that Immunity-H had higher TMB and lower levels of CNAs than Immunity-L. In addition, the markedly lower ITH in Immunity-H vs. Immunity-L supports the notion that ITH can contribute to tumor immune evasion (23, 47).

In addition to immune pathways, Immunity-H was more enriched in several cancer-associated pathways, including apoptosis, VEGF signaling, calcium signaling, MAPK signaling, and Wnt signaling pathways. We demonstrated that most of these pathways has positive associations of their enrichment with immune infiltration levels in HPV+ cervical cancer. In fact, the positive association between these pathway' enrichment and immune infiltration have been implicated in many other cancers, such as head and neck (45), gastric (48), and breast cancers [50].

Using the uncentered correlation and centroid linkage method, the TCGA network performed mRNA clustering of cervical cancers (4). There were three cervical cancer clusters identified, termed C1, C2, and C3, respectively. We found that 39.74% of Immunity-L tumors belonged to C1, compared to 9.19% of Immunity-H tumors in C1 (*P* < 0.001). It is in line with that C1 has the worst prognosis among the three clusters. In contrast, 64.37 and 26.44% of Immunity-H tumors belonged to C2 and C3, respectively, compared to 46.15 and 14.10% of Immunity-L tumors in C2 and C3, respectively. These results suggest that our immune-based subtyping method is reasonable in terms of the prognostic relevance.

By analyzing multiple datasets using both unsupervised and supervised machine learning approaches, we demonstrated that this subtyping method for HPV+ cervical cancer was stable and predictable. It suggests that this method has the application potential in clinical practice. However, further experimental and clinical validation is necessary to translate our findings into clinical practice. It should be a priority in our future studies.

## Conclusion

HPV+ cervical cancers are classified into two immune subtypes based on their immune signatures' enrichment levels. The immune subtypes have significantly different immunity, tumor proliferation potential, stemness, ITH, TMB, neoantigens, CNAs, DDR activity, and overall survival prognosis. Our data offer new understanding of tumor immunity for HPV+ cervical cancer and clinical association with its immunotherapy.

## Data availability statement

The original contributions presented in the study are included in the article/Supplementary material, further inquiries can be directed to the corresponding authors.

## Author contributions

GS: software, validation, formal analysis, investigation, data curation, and writing—review and editing. JL: software, validation, formal analysis, investigation, data curation, and visualization. SZ and FL: investigation and data curation. TZ and XZ: investigation and formal analysis. JY: investigation and supervision. XW: conceptualization, methodology, resources, investigation, writing—original draft, writing—review and editing, supervision, and project administration. All authors contributed to the article and approved the submitted version.

## Funding

This work was supported by the China Pharmaceutical University (Grant number 3150120001 to XW).

## Conflict of interest

The authors declare that the research was conducted in the absence of any commercial or financial relationships that could be construed as a potential conflict of interest.

## Publisher's note

All claims expressed in this article are solely those of the authors and do not necessarily represent those of their affiliated organizations, or those of the publisher, the editors and the reviewers. Any product that may be evaluated in this article, or claim that may be made by its manufacturer, is not guaranteed or endorsed by the publisher.
